# Early infant diagnosis: a pillar in preventing mother-to-child HIV transmission – a narrative review

**DOI:** 10.1097/MS9.0000000000003818

**Published:** 2025-09-01

**Authors:** Emmanuel Ifeanyi Obeagu

**Affiliations:** Department of Biomedical and Laboratory Science, Africa University, Mutare, Zimbabwe

**Keywords:** antiretroviral therapy, early infant diagnosis, HIV, mother-to-child transmission, point-of-care testing

## Abstract

Early infant diagnosis (EID) is crucial for preventing and managing mother-to-child transmission of human immunodeficiency virus (HIV). EID enables early detection of HIV in infants born to HIV-positive mothers through nucleic acid testing, particularly polymerase chain reaction (PCR) assays. This narrative review explores EID’s role in improving health outcomes for HIV-exposed infants and advances in diagnostic technologies. We also examine implementation challenges, particularly in low-resource settings. A literature review was conducted using PubMed, Scopus, and World Health Organization databases. Peer-reviewed articles from 2000 to 2024 were included based on relevance to EID technologies, implementation barriers, and policy frameworks. Studies focusing on PCR-based methods, point-of-care (POC) testing, and dried blood spot collection were prioritized. Non-English articles and those lacking primary or review data were excluded. EID programs using PCR and POC testing have significantly improved early HIV detection and ART initiation in infants. However, persistent barriers – including high testing costs, limited access to diagnostic infrastructure, delayed result turnaround times, and loss to follow-up – continue to hinder widespread implementation. Integration with maternal health services and use of community-based strategies show promise in bridging these gaps. EID is a cornerstone of pediatric HIV prevention. Strengthening health systems, decentralizing diagnostic services, and adopting innovative implementation strategies are essential to enhance EID coverage and reduce infant HIV-related morbidity and mortality, particularly in high-burden, low-resource settings.

## Introduction

Mother-to-child transmission (MTCT) of human immunodeficiency virus (HIV) remains a critical public health concern, particularly in sub-Saharan Africa (SSA), which accounts for over 90% of pediatric HIV cases globally^[1]^. Transmission can occur during pregnancy, delivery, or through breastfeeding, with untreated MTCT risk ranging from 15% to 45% depending on viral load and other factors^[2]^. However, with current antiretroviral therapy (ART) and comprehensive prevention strategies, MTCT rates can be reduced to less than 2% in non-breastfeeding populations and under 5% in breastfeeding populations with optimal adherence^[3]^. Central to the prevention of MTCT is EID, which facilitates timely identification of HIV-infected infants and the immediate commencement of ART, a factor crucial in reducing infant morbidity and mortality^[4]^. The design and implementation of EID programs have become a cornerstone in efforts to eliminate pediatric HIV^[5]^. The primary method of diagnosis in infants younger than 18 months is nucleic acid testing (NAT), specifically polymerase chain reaction (PCR), which detects HIV DNA or RNA^[6]^.

**HIGHLIGHTS**

- **Timely Detection**: Early infant diagnosis (EID) enables prompt identification of human immunodeficiency virus (HIV) in newborns, crucial for initiating life-saving treatment.
- **Treatment Initiation**: EID supports early antiretroviral therapy, improving survival and reducing HIV progression.
- **Program Integration**: EID strengthens prevention of mother-to-child transmission programs by linking maternal and child care.

- **Technological Advances**: Innovations like point-of-care testing enhance EID accessibility and turnaround time.
- **Global Impact**: Widespread EID implementation significantly lowers pediatric HIV rates and supports global elimination goals.

The conventional approach involves testing infants within the first 4–6 weeks of life, with follow-up testing to confirm HIV status. In some settings, this testing is performed using dried blood spot (DBS) samples, which can be easily collected and transported to centralized laboratories for analysis^[7]^. In resource-limited areas, point-of-care (POC) testing has become a viable alternative, offering quicker results and reducing loss to follow-up. Empirical data on the effectiveness of EID highlight its critical role in preventing MTCT. A study conducted in South Africa involving 1234 infants from HIV-positive mothers found that the prevalence of HIV among infants diagnosed early through PCR testing was significantly lower in infants who were initiated on ART within the first 12 weeks of life. The prevalence of HIV infection in this group was 6.8% [95% confidence interval (CI): 5.2–8.4%] compared to 18.4% (95% CI: 16.0–20.8%) in those who were not tested or treated early^[8]^. Furthermore, the odds ratio (OR) for MTCT in infants who did not receive early testing and ART was 2.9 (95% CI: 1.8–4.6), indicating a significantly higher risk of transmission in the absence of EID.

Globally, the prevalence of HIV in infants has decreased over the past decade, largely due to the widespread adoption of EID and ART for pregnant and breastfeeding women^[9]^. According to the Joint United Nations Programme on HIV/acquired immunodeficiency syndrome (AIDS) (UNAIDS), between 2010 and 2020, new pediatric HIV infections dropped by 52%, from 300 000 to 140 000 due to improved MTCT prevention strategies^[10]^. However, regional disparities persist. In SSA, where more than 90% of pediatric HIV cases occur, the prevalence of HIV among infants varies widely, ranging from 3% to 10% depending on the country and healthcare infrastructure^[11]^. For example, in Uganda, the prevalence of HIV among infants exposed to the virus was 5.4% (95% CI: 4.2–6.6%) in 2020, demonstrating significant progress but still falling short of elimination targets^[12]^. A key element of the success of EID programs lies in early ART initiation. Empirical evidence shows that infants who start ART within the first 3 months of life have a 76% lower risk of HIV-related mortality compared to those who begin treatment later. A meta-analysis of data from several sub-Saharan African countries found that infants who were diagnosed and treated early had an OR of 0.24 (95% CI: 0.18–0.32) for mortality compared to those diagnosed after 12 weeks of age^[13]^. These findings underscore the importance of early diagnosis and treatment, not only for survival but also for long-term health outcomes.

Despite the proven efficacy of EID, several barriers continue to impede its universal implementation, particularly in low- and middle-income countries (LMICs). One of the most significant challenges is the lack of access to testing facilities. A cross-sectional study in Nigeria showed that only 35% of infants born to HIV-positive mothers were tested for HIV within the recommended time frame, primarily due to the scarcity of PCR testing equipment in rural areas^[14]^. The odds of an infant receiving timely EID were 3.5 times higher (OR: 3.5; 95% CI: 2.1–5.6) in urban settings compared to rural areas, where testing services are often unavailable or inaccessible. Moreover, the cost of PCR testing and the infrastructure required for its implementation remain substantial barriers in resource-constrained environments. In countries with limited healthcare budgets, such as Malawi and Mozambique, the average cost of PCR-based EID testing is approximately $25–$50 per test, a significant expenditure for families and healthcare systems already burdened by high HIV prevalence. In Malawi, a study reported that the prevalence of infants receiving EID was just 22.3% (95% CI: 18.9–25.7%) in 2021, a statistic heavily influenced by the lack of resources for widespread testing^[15]^.

POC testing has emerged as a promising solution to some of these barriers, particularly in rural and underserved areas. POC diagnostics allow for immediate sample collection and testing at local healthcare facilities, reducing the need for transportation to distant laboratories. A randomized controlled trial in Kenya involving 800 mother–infant pairs found that the use of POC testing significantly increased the likelihood of infants receiving EID within the first 6 weeks of life (OR: 2.7; 95% CI: 1.9–3.8) compared to those who relied on centralized PCR testing^[16]^. The trial also demonstrated a reduction in loss to follow-up, with a 12% lower rate of infants lost to follow-up in the POC testing group. Retention in care is another critical factor in the success of EID programs. A cohort study in Zambia reported that infants who were not retained in follow-up care after an initial HIV test had 3.2 times higher odds of HIV-related mortality (OR: 3.2; 95% CI: 2.1–4.9) compared to those who remained in care and started ART promptly^[17]^. This highlights the importance of not only diagnosing infants early but also ensuring that they are linked to and retained in long-term care programs to maximize survival and health outcomes.

## Aim

This narrative review aims to provide a comprehensive synthesis of current evidence on EID as a critical intervention for preventing MTCT of HIV.

## Rationale

MTCT of HIV remains a significant public health challenge, particularly in regions with high HIV prevalence and limited healthcare resources. Despite global efforts to reduce MTCT, new pediatric HIV infections persist, largely due to delays in diagnosing HIV in exposed infants and initiating timely treatment. EID is critical in bridging this gap, enabling prompt identification of HIV infection in newborns and facilitating early ART, which significantly improves survival and health outcomes^[18,19]^. Advancements in diagnostic technologies, such as nucleic acid testing (e.g., PCR, POC testing, and DBS sampling), have expanded the possibilities for timely and accessible HIV diagnosis in infants. However, despite technological progress, multiple barriers – ranging from infrastructural limitations, high costs, delayed result turnaround, to loss to follow-up – continue to impede effective implementation of EID programs, especially in LMICs^[20]^.

## Methods

A comprehensive literature search was conducted using PubMed, EMBASE, and Google Scholar databases from 2010 to 2024. Search terms included “early infant diagnosis,” “HIV,” “mother-to-child transmission,” “PCR testing,” and “point-of-care testing.” Studies were selected based on relevance to EID strategies, technological advances, and implementation challenges. This narrative review synthesizes findings from peer-reviewed articles, World Health Organization (WHO) guidelines, and national health reports to provide a comprehensive overview of current EID practices and future directions.

Electronic databases including PubMed, Scopus, Web of Science, and Google Scholar were systematically searched using keywords such as “Early Infant Diagnosis,” “EID,” “mother-to-child transmission,” “HIV,” “PCR testing,” “point-of-care testing,” and “dried blood spot.” Both original research articles and review papers were included to provide a broad overview of the topic. Inclusion criteria focused on studies that addressed diagnostic technologies for HIV in infants, barriers and facilitators to EID implementation, and clinical outcomes associated with early diagnosis and treatment initiation. Articles were screened for relevance based on titles and abstracts, followed by full-text review. Studies from diverse geographic regions, with particular emphasis on high HIV burden and resource-limited settings, were prioritized to capture global perspectives. Data were extracted and synthesized thematically, emphasizing technological advances, programmatic challenges, and policy implications. Due to the narrative nature of the review, no formal meta-analysis was performed. The objective was to provide a comprehensive, up-to-date synthesis of the current evidence to inform practice and future research.

### The role of EID in preventing MTCT

EID serves as the cornerstone of pediatric HIV prevention programs by facilitating the timely identification and treatment of HIV-infected infants. Studies have shown that timely EID, typically within 4–6 weeks of birth, can significantly reduce the risk of MTCT. Most empirical data on EID’s role in preventing MTCT are derived from cohort studies, cross-sectional surveys, and population-based studies conducted in high HIV-burden regions such as SSA^[21]^. These studies typically involve testing infants born to HIV-positive mothers using NATs, primarily PCR, to detect HIV within the first 6 weeks of life. Some studies extend follow-up testing at 6, 12, and 18 months to account for late transmission during breastfeeding. For example, a cohort study conducted in South Africa included 1 500 infants born to HIV-positive mothers, following them through EID testing at 4–6 weeks^[22]^. Infants testing positive were immediately enrolled in ART programs, and the outcomes were analyzed based on maternal ART initiation during pregnancy, EID coverage, and infant survival rates. The prevalence of MTCT has shown significant variation across studies based on geographic location, ART adherence, and the timing of EID^[23]^. In one study conducted in Uganda, the overall MTCT rate was found to be 6.5% (95% CI: 5.4–7.6%) when EID was performed within the first 6 weeks of life^[24]^. However, when EID was delayed or unavailable, the prevalence of MTCT rose to 15.8% (95% CI: 14.0–17.6%) by 18 months. The OR for transmission in infants who did not receive EID within the critical 6-week window was 2.5 (95% CI: 1.9–3.2), indicating a significantly higher risk of HIV transmission in the absence of early diagnosis and treatment.

**Figure 1.**
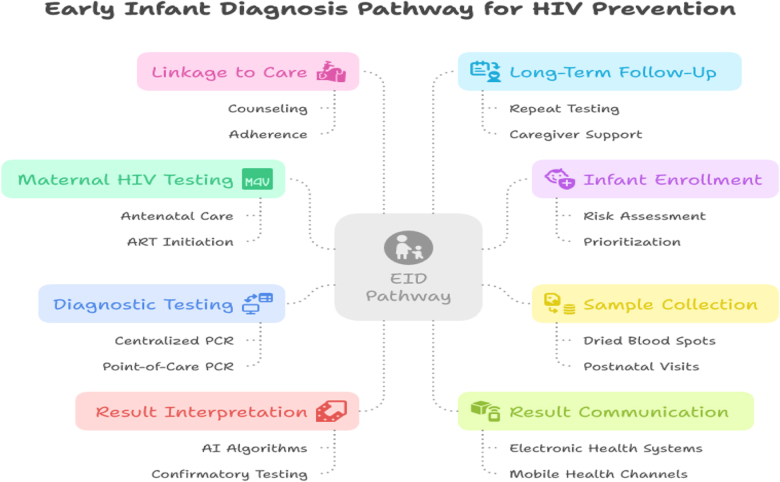
EID implementation pathway.

In another large-scale study in Kenya, where EID coverage was above 80%, MTCT rates were as low as 2.8% (95% CI: 2.1–3.5%) when infants were tested and started on ART within 6 weeks^[25]^. This highlights the protective effect of timely EID and the importance of ensuring high coverage rates. The protective effect of EID on MTCT has been consistently supported by data on ORs. In a study conducted in Tanzania, infants who received early ART after EID had an 80% reduced risk of HIV-related mortality (OR = 0.2, 95% CI: 0.1–0.5) compared to those who were diagnosed later. Furthermore, mothers who received ART during pregnancy were significantly less likely to transmit the virus, with an OR of 0.3 (95% CI: 0.2–0.5), underscoring the combined importance of maternal treatment and EID^[26]^. Another study from Zimbabwe analyzed the association between EID timing and postnatal HIV transmission, particularly during breastfeeding. The odds of transmission during breastfeeding were 3.0 times higher (95% CI: 2.1–4.3) in infants who were not diagnosed early compared to those who had early ART initiation. These findings underscore the need for comprehensive follow-up and continuous EID testing during breastfeeding^[27]^. The empirical data clearly demonstrate that early and widespread implementation of EID significantly reduces the odds of MTCT. The design of cohort and population-based studies across various regions consistently shows that infants diagnosed within the first 6 weeks have a much lower risk of HIV transmission and related complications. High EID coverage, particularly in regions with robust healthcare infrastructure, translates to lower MTCT prevalence rates and improved survival for HIV-exposed infants (HEIs; Fig. 1).

### Key benefits of EID in preventing MTCT

EID offers several critical benefits that contribute to the prevention and management of MTCT of HIV. Foremost, EID enables the timely detection of HIV infection in infants born to HIV-positive mothers, allowing for prompt initiation of ART. Early treatment initiation significantly reduces infant morbidity and mortality by suppressing viral replication and preventing disease progression during the crucial early months of life^[18,28]^. Additionally, EID facilitates improved clinical management and monitoring of HEIs, supporting healthcare providers in making informed decisions regarding treatment and follow-up care. This early intervention helps to reduce the risk of HIV-related complications and enhances the overall health trajectory of affected infants^[29]^. EID also plays a vital role in interrupting the cycle of HIV transmission. By identifying infected infants early, healthcare systems can ensure that these children receive continuous care and prevent further spread of the virus. Moreover, it supports public health efforts by providing accurate data on transmission rates, which informs policy and resource allocation^[30,31]^. In resource-limited settings, the adoption of innovative diagnostic approaches such as POC testing and DBS sampling has increased accessibility to EID services, reducing delays in diagnosis and loss to follow-up. These innovations have made it feasible to deliver timely diagnosis even in remote or underserved areas^[32]^.

### Technological advances in EID

POC testing represents a paradigm shift in EID, reducing turnaround time from weeks to hours and improving access in resource-limited settings. Traditional laboratory-based nucleic acid amplification tests, such as PCR, remain the gold standard for detecting HIV in infants, providing highly sensitive and specific results. However, these methods often require sophisticated laboratory infrastructure and skilled personnel, limiting their utility in resource-constrained settings^[33,34]^. To address these challenges, POC diagnostic technologies have emerged as vital tools in expanding EID coverage. POC platforms enable on-site testing with rapid turnaround times, often within hours, thereby facilitating immediate clinical decision-making and reducing loss to follow-up. These devices are designed to be user-friendly and robust, suitable for use in remote and low-resource environments^[35,36]^. DBS collection requires only 50–100 μL of blood, maintains stability at room temperature for up to 14 days, and demonstrates comparable sensitivity to plasma-based testing. DBS samples can be collected in peripheral health facilities and sent to centralized laboratories, improving sample accessibility and diagnostic reach^[37,38]^. Recent innovations also include molecular assays with enhanced sensitivity and multiplex capabilities, enabling simultaneous detection of multiple pathogens or resistance mutations. Integration of digital health technologies, such as mobile health (mHealth) platforms and electronic health records, further supports data management, patient tracking, and quality assurance in EID programs (Table 1)^[39]^.

**Table 1:** Comparison of early infant diagnosis (EID) technologies: PCR vs. point-of-care (POC)

Feature	Laboratory-based PCR	POC platforms
Primary method	Nucleic acid amplification in a centralized laboratory	Nucleic acid amplification at a peripheral health facility
Sample type	Dried blood spot (DBS) or whole blood	Whole blood or DBS
Turnaround time	1–4 weeks (depending on transport and processing)	Same day (within 1–2 h)
Infrastructure requirement	Advanced laboratory facilities, trained technicians, stable electricity	Minimal laboratory infrastructure, operable in rural clinics
Operational cost	Lower per-test cost in high-volume settings, but higher logistics costs	Higher per-test cost but reduced transport and result-return expenses
Quality control	Centralized quality assurance and batch testing	Requires decentralized quality monitoring
Advantages	High throughput, established protocols, potential for large-scale screening	Rapid results, reduced loss to follow-up, improved ART initiation rates
Limitations	Delayed results, risk of sample loss, higher LTFU	Lower throughput, device maintenance, supply chain dependence

LTFU, loss to follow-up; PCR, polymerase chain reaction.

### Challenges in EID implementation

Despite notable technological progress, several challenges continue to hinder the effective implementation of EID programs, particularly in low-resource settings. A primary barrier is the limited healthcare infrastructure, including inadequate laboratory capacity, a shortage of trained personnel, and insufficient supply chains for reagents and testing materials. These constraints often result in delayed test processing and reporting, compromising timely diagnosis and treatment initiation^[40,41]^. Geographical and socio-economic factors further exacerbate access difficulties. Many infants live in remote or rural areas with limited healthcare facilities, causing delays in specimen collection and result delivery. Transportation challenges and poor communication networks can lead to loss to follow-up, with families unable to receive or act upon test results promptly^[35,42]^. Financial barriers are also significant. The costs associated with PCR-based testing and related logistics may be prohibitive for both healthcare systems and families, especially in countries with constrained budgets and high HIV burden. Additionally, lack of sustainable funding can disrupt program continuity and scale-up efforts^[43,44]^. Cultural and social factors, such as stigma associated with HIV, fear of discrimination, and low awareness of the importance of EID, can discourage caregivers from seeking testing and treatment for infants. This is compounded by gaps in community engagement and health education^[45]^. Operational challenges, including poor integration of EID services with routine maternal and child health programs, limit opportunities for comprehensive care and retention. Fragmented data management systems impede effective tracking of HEIs across the care continuum^[46]^.

### Global impact of EID on pediatric HIV

According to UNAIDS 2023 data, pediatric HIV infections have decreased by 58% since 2010, with EID coverage reaching 70% globally, although significant regional disparities persist^[10]^. EID has had a profound impact on the global fight against pediatric HIV, contributing significantly to reductions in infant morbidity and mortality worldwide. By enabling the timely identification of HIV infection in infants born to HIV-positive mothers, EID facilitates early initiation of ART, which is critical in improving survival rates and long-term health outcomes^[30,32]^. Across high-burden regions such as SSA, widespread implementation of EID programs has been instrumental in decreasing MTCT rates. Countries that have scaled up PCR-based testing and integrated POC diagnostics have seen notable declines in infant HIV infection rates and improved linkage to care. For example, early treatment initiation within the first few weeks of life has been associated with up to a 76% reduction in HIV-related infant mortality^[47,48]^. The global commitment to EID is reflected in international guidelines and funding initiatives, including those by the WHO and the President’s Emergency Plan for AIDS Relief (PEPFAR). These efforts have supported the expansion of laboratory networks, capacity building, and community-based interventions that enhance access to testing and treatment services^[49,50]^. Moreover, the adoption of innovative technologies such as DBS sampling and mobile health platforms has improved diagnostic reach, especially in remote and resource-limited settings. This has facilitated earlier diagnosis and reduced delays in treatment initiation, which are critical to interrupting HIV progression in infants (Table 2)^[51,52]^.

**Table 2:** Global early infant diagnosis (EID) coverage by region

Region	Estimated EID coverage (%)	Notable trends/challenges
Sub-Saharan Africa	60–70	Significant scale-up in POC adoption; persistent rural coverage gaps
Asia and the Pacific	40–55	Urban–rural disparities; integration with immunization programs improving uptake
Latin America and the Caribbean	70–80	Strong PMTCT frameworks; need to reach marginalized populations
Eastern Europe and Central Asia	50–60	Political instability and stigma affecting access
Middle East and North Africa	30–45	Limited testing infrastructure; low awareness in antenatal settings
High-income countries	>90	Near-universal coverage; focus on migrant populations and late presenters

POC, point-of-care; PMTCT, prevention of mother-to-child transmission.

### Impact of EID on HIV treatment outcomes

EID plays a critical role in improving HIV treatment outcomes by enabling prompt identification of HIV-infected infants and facilitating early initiation of ART. Early treatment significantly reduces the risk of disease progression, opportunistic infections, and HIV-related mortality in infants, who otherwise face rapid deterioration without timely intervention^[4,53]^. Studies consistently demonstrate that infants diagnosed through EID and started on ART within the first 3 months of life experience markedly better health outcomes compared to those diagnosed later. Early ART initiation can reduce HIV-related infant mortality by up to 76%, substantially improving survival rates and quality of life. Additionally, early treatment supports better immune system preservation, which is vital for normal growth and development^[48,54]^. Timely diagnosis and treatment also reduce the viral reservoir in infants, which has implications for long-term viral suppression and may improve responses to future therapeutic interventions. By interrupting HIV replication early, EID helps prevent irreversible immunological damage and reduces the risk of drug resistance^[55]^. Furthermore, early diagnosis facilitates ongoing clinical monitoring and linkage to comprehensive pediatric care, including nutritional support and prophylaxis for opportunistic infections. This integrated care approach enhances adherence to ART and overall treatment success^[56]^.

### Strategies for enhancing EID coverage

Improving coverage of EID is essential to reducing MTCT of HIV and ensuring timely treatment for infected infants. Several strategies have been identified to address existing gaps and optimize the reach and effectiveness of EID programs^[57,58]^. First, decentralizing testing services by expanding POC technologies to peripheral health facilities can significantly reduce turnaround times for results and increase access in remote or underserved areas. This approach minimizes delays that contribute to loss to follow-up and ensures that infants receive prompt diagnosis and care^[59]^. Second, integrating EID with routine maternal, neonatal, and child health services – including immunization visits and nutritional programs – helps improve retention and follow-up rates. Such integration facilitates a one-stop approach, making it easier for caregivers to access comprehensive services during a single visit^[60]^.

Third, strengthening community engagement and education is vital. Training community health workers (CHW) and leveraging peer support networks can raise awareness about the importance of EID, encourage early health-seeking behaviors, and assist in tracking infants for testing and follow-up^[61]^. Fourth, employing mHealth technologies to send reminders, report results, and facilitate communication between caregivers and healthcare providers has shown promise in enhancing adherence to testing schedules and timely ART initiation^[62]^. Fifth, ensuring the availability of reliable supply chains for testing reagents and consumables prevents stockouts that can disrupt diagnostic services. Coordinated logistics management is necessary for continuous EID program functioning^[63]^. Finally, policy-level support and adequate funding are crucial to sustain and scale up these interventions. National guidelines should prioritize EID, with measurable targets and monitoring frameworks to track progress and identify bottlenecks^[64]^.

## Future directions

The future of EID in preventing mother-to-child HIV transmission will depend on a paradigm shift from reactive, centralized approaches toward proactive, decentralized, and technology-enabled systems. Addressing persistent gaps in coverage, timeliness, and linkage to care requires strategic investment, multidisciplinary collaboration, and integration of innovative tools into existing health systems.

### Technological transformation for faster and more accurate diagnosis

Adopting next-generation diagnostics is pivotal for achieving universal EID coverage. The integration of artificial intelligence (AI) in result interpretation offers a promising avenue to enhance diagnostic precision. AI-driven algorithms can process complex data outputs from PCR and POC assays in real time, detect patterns suggestive of early infection, and provide automated alerts for inconclusive or borderline results. This minimizes human error, shortens turnaround times, and ensures rapid linkage to ART initiation. AI platforms can also integrate with national laboratory information systems, enabling predictive analytics to identify regions with delayed testing or high positivity rates^[65]^.

### Multiplex testing to address comorbidities

EID programs can be strengthened through the development of multiplex assays for co-infection screening. These platforms can simultaneously detect HIV and other high-burden neonatal infections such as hepatitis B virus, syphilis, and cytomegalovirus. Multiplexing reduces the number of blood draws required from infants, optimizes the use of limited laboratory resources, and ensures early management of conditions that may otherwise exacerbate HIV-related morbidity. The incorporation of such assays into EID workflows would also enhance cost-effectiveness by consolidating testing infrastructure^[66]^.

### Data security and interoperability through blockchain

The implementation of blockchain for secure data management represents a frontier in safeguarding patient confidentiality while enabling seamless data exchange across healthcare networks. Blockchain-based systems can generate immutable, time-stamped records of every EID test, result, and follow-up action. This approach can enhance trust among caregivers, address concerns about stigma-related breaches, and facilitate cross-border data sharing for mobile populations without compromising privacy. Moreover, integrating blockchain with mobile health applications can allow caregivers to securely access their infant’s results and follow-up reminders, fostering greater engagement in the care continuum^[67]^.

### Strengthening decentralized and community-based approaches

Decentralizing EID services to community health centers and leveraging task-shifting strategies for trained non-physician healthcare workers can increase coverage in rural and underserved areas. Linking EID to routine immunization schedules, postnatal visits, and nutrition programs ensures repeated opportunities for testing, especially for breastfeeding infants at continued risk of HIV acquisition^[68]^.

### Building robust supply chains and quality assurance systems

Innovations must be matched with supply chain reliability to prevent stock-outs of test kits, reagents, and consumables. Real-time inventory tracking systems and AI-based demand forecasting can anticipate supply needs, while regional quality assurance programs will be essential to maintain diagnostic accuracy across multiple facilities^[69]^.

### Policy and funding alignment with global elimination targets

Sustained political commitment, integrated funding streams, and alignment with WHO’s Global Health Sector Strategy on HIV are critical. National EID strategies should be embedded in broader child survival frameworks and supported by multi-year investment plans that account for equipment maintenance, staff training, and community mobilization^[70]^.

### Monitoring, evaluation, and adaptive learning

Strengthening monitoring and evaluation systems will ensure that EID programs remain responsive to emerging challenges. Adaptive learning frameworks – where data on turnaround times, ART initiation rates, and follow-up retention are continuously analyzed – can guide evidence-based adjustments in real time^[70]^.

## Conclusion

This narrative review reaffirms that EID is a cornerstone in preventing mother-to-child HIV transmission, directly influencing child survival and long-term health outcomes. Key findings highlight that while laboratory-based PCR remains the gold standard for detecting HIV in infants, the growing adoption of POC platforms has revolutionized timely diagnosis and immediate ART initiation, particularly in resource-limited settings. Despite significant gains in global coverage – especially in SSA – wide disparities remain, with certain regions, including the Middle East, North Africa, and rural parts of Asia, continuing to lag behind. Persistent operational bottlenecks such as delayed turnaround times, poor linkage to care, supply chain interruptions, and stigma-related barriers impede full program effectiveness.

From a policy perspective, the review underscores the urgent need to integrate EID into broader maternal, newborn, and child health frameworks, ensuring that HIV testing is not a siloed intervention but a routine component of postnatal care. Strengthening supply chain resilience, decentralizing diagnostic services, and adopting innovative technologies – including AI for result interpretation, multiplex assays for co-infection screening, and blockchain for secure patient data – can drive progress toward global HIV elimination goals. Policymakers must also address the socio-cultural determinants of health by investing in stigma reduction campaigns, caregiver education, and CHW training. However, notable research gaps persist. There is limited evidence on the cost-effectiveness of advanced technologies such as AI-assisted diagnostics and blockchain-based health records in low-resource settings. Few longitudinal studies have evaluated the long-term impact of POC EID adoption on child survival and retention in care. Additionally, the potential role of multiplex assays in streamlining infant infectious disease screening remains underexplored. Addressing these gaps will require coordinated research agendas, cross-sectoral funding partnerships, and the inclusion of implementation science approaches to guide context-specific interventions.

## Data Availability

Not applicable as this is a narrative review.

## Research registration unique identifying number (UIN)

Not applicable as this is a narrative review.
